# Factors Influencing Marksmanship in Police Officers: A Narrative Review

**DOI:** 10.3390/ijerph192114236

**Published:** 2022-10-31

**Authors:** Vini Simas, Ben Schram, Elisa F. D. Canetti, Danny Maupin, Robin Orr

**Affiliations:** Tactical Research Unit, Bond Institute of Health and Sport, Bond University, Robina, QLD 4226, Australia

**Keywords:** shooting accuracy, law enforcement personnel, grip strength, load carriage

## Abstract

Law enforcement officers routinely face unpredictable scenarios that may threaten the public, their colleagues, or themselves. In such situations, officers may be required to use firearms, with shooting accuracy becoming crucial. This literature review aimed to identify, synthesize, and report on research investigating factors that affect marksmanship in law enforcement personnel. A systematic search of seven databases was undertaken following the Preferred Reporting Items for Systematic Reviews and Meta-Analysis (PRISMA) approach. From an initial 5396 identified studies, 23 met the eligibility criteria. The key findings from these papers were: (1) physical exertion does not appear to decrease shooting performance, especially at close range (<10 m); (2) tactical load carriage does not appear to decrease shooting performance; in fact, it has been reported by officers as improving performance (likely due to training specificity); (3) the physical capability of officers may be of importance, notably grip strength, which the volume of evidence suggests is positively correlated with marksmanship; (4) anxiety imparted through high-stress scenarios negatively impacts shooting performance, but training under stress may counteract this factor, albeit for a short period. Together, these factors appear to have a trainability component where increased specific and realistic training can improve shooting accuracy, time, and precision, especially in high-stress situations.

## 1. Introduction

Law enforcement personnel may encounter situations that pose a threat to their safety or the safety of their co-workers or fellow citizens [1]. In such situations, felons may be well-equipped with firearms and prepared to use them to avoid arrest [2]. The officer’s decision and action to employ their sidearm typically occurs in situations of high anxiety, a trait known to negatively impact on marksmanship [3], and under time-compressed conditions [4] where failure could ultimately result in possible harm to the officer, suspect or the general public [5,6,7]. As such, for a law enforcement officer, who may rarely, if ever, use their firearm [8], firearm technical and tactical skills are of high importance.

Considering the importance of firearm proficiency and accuracy, the poor marksmanship of police officers has led to officers missing their target when engaging an armed suspect [9] and has led to bystander fatalities [10]. In a study of the Dallas Police Department of officer-involved shootings from 2003 to 2017, 54% of firearm discharge events resulted in a hit [8]. Furthermore, when considering total rounds fired, hit rate dropped to 35%, with 231 rounds missing the intended target [8]. These findings are supported by reporting on the Metropolitan Police Department in Las Vegas, which identified a hit rate accuracy ranging from 23% to 52% over the period of 2008–2015 [11,12]. Statistics from the New York Police Department show that the average hit rate was 30% in situations where gunfire was not returned and 18% for officers involved in a gunfight [13]. These statistics highlight the requirement to invest in improving officer marksmanship capability, especially under high-threat situations where the threat posed by the offender has led to a further decrease in officer marksmanship [8].

Prior to devising an optimal marksmanship training program and governing assessments and standards, factors that impact an officer’s marksmanship require consideration. As such, this review aimed to identify, examine, and synthesize peer-reviewed published literature reporting on factors affecting marksmanship in law enforcement personnel.

## 2. Materials and Methods

To address the aims of the present study, a narrative synthesis of the literature was undertaken, guided by the Preferred Reporting Items for Systematic Reviews and Meta-Analysis (PRISMA) statement [14].

### 2.1. Eligibility Criteria

To be included in this review, studies were required to meet all the inclusion criteria and fail to meet any exclusion criteria (Table 1), regardless of publication date.

### 2.2. Information Sources and Search

A systematic search was conducted in March 2022 to identify relevant publications to inform the narrative synthesis in the following databases: PubMed, Cumulative Index to Nursing and Allied Health Literature (CINAHL), Embase, SPORTDiscus, Scopus, Cochrane, and Web of Science. Additionally, reference lists of included articles were screened to identify potentially relevant studies. The search strategy included search terms relative to ‘police’ and ‘marksmanship’ and was adapted according to the requirements of each database. The search strategy used in PubMed is provided in Table 2 as an example, and full search strategies for other databases are provided in Supplementary Table S1.

### 2.3. Study Selection, Data Collection and Synthesis

All retrieved results were imported into a reference management software (EndNote, version X9.2, Clarivate Analytics, Philadelphia, PA, USA), where duplicates were removed. After screening titles and abstracts, full texts of potentially relevant studies were retrieved. Eligibility criteria (Table 1) were then applied, and relevant studies were retained. The selection process was documented in a PRISMA flow diagram [14]. For all included studies, the following data were extracted into a spreadsheet: authors, year of publication, study design, population, settings, intervention/comparison (if appropriate), details relevant to marksmanship assessment, variables investigated, and summary of statistical analyses. A narrative synthesis was then conducted, reporting information relevant to address the aims of the present study.

## 3. Results

**Figure 1:**
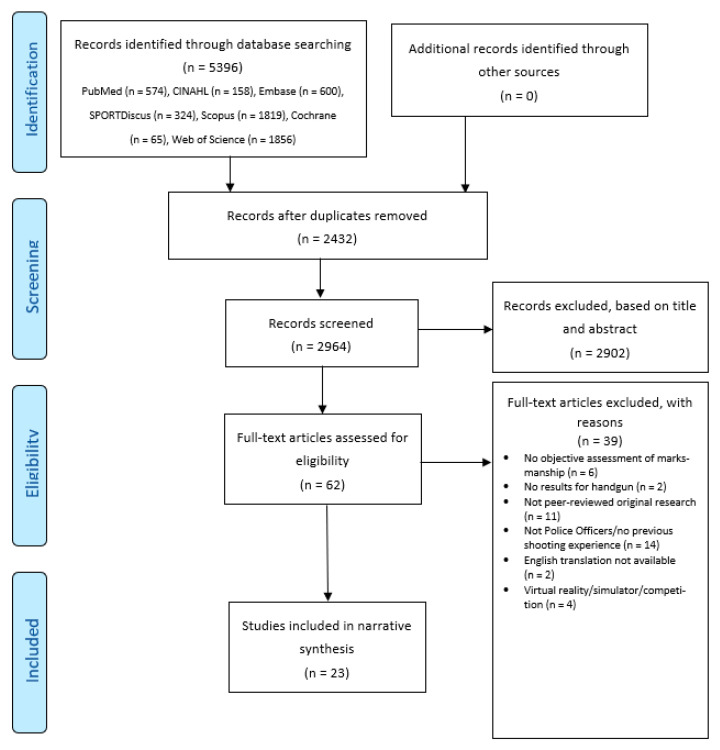
PRISMA flow chart diagram outlining the results of the search, screening, and selection process.

The database searches retrieved 5396 references, including 2432 duplicates (Figure 1). A total of 2964 titles and abstracts were screened, and 2902 references were considered not relevant to this review. Full texts of 62 studies were assessed against the eligibility criteria (Table 1), and 23 research papers were included in the review [2,3,5,7,15,16,17,18,19,20,21,22,23,24,25,26,27,28,29,30,31,32,33].

### 3.1. Study Selection, Data Collection and Synthesis

The included studies were conducted in Australia [7,15,18,30,31], Brazil [22], Canada [5,16], Czech Republic [2], Netherlands [19,24,25,27,28,32], Turkey [3], and the USA [17,20,21,23,26,29,33]. Of the 23 included studies, ten were cross-sectional studies [3,5,15,16,20,24,25,27,29,31], seven were quasi-experiments [2,17,21,22,23,32,33], two were randomized controlled trials [19,28], two were crossover trials [18,30], and two were retrospective cohort studies [7,26]. Individuals assessed in the studies were regular police officers/recruits [3,5,7,15,16,17,19,20,21,23,25,26,27,28,29,31,32] and members of specialist units [2,18,22,30,33]. One study assessed both police officers and specialist officers [24]. Data extracted for each study in relation to the population, research variables, and research results of selected studies are provided in Table 3. Additional data detailing the country, any marksmanship interventions, and marksmanship assessments employed can be found in Supplementary Table S2.

### 3.2. Physical and Physiological Factors

Fifteen studies [2,3,5,7,15,16,17,18,21,22,23,26,30,31,33] assessed physical and/or physiological factors and their relationships with marksmanship (Table 3). Anderson and Plecas [5] reported significant and positive correlations between shooting scores and Police Officer Physical Abilities Test (POPATS), dominant hand grip strength, combined hand grip strength, forearm girth, and second finger ray length; however, the results were found when male and female subjects were analyzed together. In the sex-specific groups, no correlations were found. Copay and Charles [21] assessed the relationship between grip strength training and marksmanship. Although no significant difference was found in grip strength pre- and post-intervention, the authors reported a statistically significant positive relationship between grip strength and shooting scores. Likewise, in another study assessing the influence of grip strength, among other factors, Kayihan et al. [3] reported a statistically significant positive correlation between grip strength and shooting performance. The authors also reported statistically significant results for change in heart rate, mean and maximum heart rate during shooting, coordination (assessed by the Alternate Hand Wall Toss Test), balance (assessed by the Stork Balance Standing Test), biceps circumference, femur diameter, wrist circumference, and flexibility [3]. Similarly, Orr et al. [7] also reported a statistically significant positive association between grip strength and marksmanship. In line with the aforementioned studies, Brown et al. [16] assessed performance on a Police Pistol Qualification (PPQ) shoot according to grip strength and found that lower grip strength was associated with poorer results. This finding was more evident when comparing officer sex and marksmanship, with female officers having lower grip strength and achieving lower results on the shooting assessment [16].

By contrast, in a recent study by Orr et al. [31], no association was found between shooting performance and grip strength. Moreover, the authors found a negative trend between marksmanship and grip strength in male officers, although this association was not statistically significant. However, the opposite was found in female officers, with marksmanship performance increasing with increased grip strength [31]. Similar results were reported by Muirhead et al. [26] who assessed three different scenarios (static shooting, dynamic scenario, and positive identification scenario; Supplementary Table S2) and reported a significant negative correlation between grip strength and marksmanship points (higher scores being better performance) in the dynamic scenario [26]. Their study also reported significant associations between marksmanship and the 20 m multi-stage fitness test (in the static shooting scenario) and leg/back strength (in the positive identification scenario; Table 3) [26].

Billich et al. [2] investigated the effect of physical exertion on shooting performance in a study where participants were exposed to maximum heart rate while exercising on a treadmill until exhaustion, followed by a shooting trial (Supplementary Table S2), and the authors reported a negative effect of physical exertion on shooting accuracy (Table 3). Contrarily, in a similar study, Brown et al. [17] reported that marksmanship (shooting accuracy and precision) was not negatively affected by physical exertion, achieved by exercising on a cycle ergometer until reaching 85% HR_max_ or exhaustion (Supplementary Table S2). A similar result was reported in another comparable study, conducted by Do Nascimento Neto et al. [22], where participants shot after completing a 297 m obstacle course, achieving 85% HR_max_ (Supplementary Table S2), and the authors found that physical exertion did not affect shooting performance, time, or efficiency (Table 3).

Hornsby et al. [23] investigated the influence of heart rate biofeedback intervention on shooting performance (Supplementary Table S2). In their study, a group of officers was exposed to the intervention, and their results were compared to a control group (i.e., no intervention) [23]. Throughout the investigation, heart rate, breathing rate, alertness, and sleep were similar between the groups, and so were the marksmanship accuracy and time to complete the shooting assessment [23]. The authors concluded that using heart rate biofeedback does not benefit the shooting performance [23].

Carbone et al. [18] assessed the effect of tactical load on marksmanship in specialist police officers in two scenarios: static and mobile trials (Supplementary Table S2). A significant difference was found when assessing horizontal dispersion (distance between the two farthest horizontally displaced falls-of-shot) in the static trial, favoring the tactical loaded condition. Tactical load was also assessed by Orr et al. [30], in a similar study. In their investigation, the authors failed to find any correlation between tactical load and marksmanship, although the specialist police officers in the study reported a subjective perception of marksmanship improvement when tactically loaded [30]. This tactical load condition was also investigated in the study by Thomas et al. [33], where no difference in shooting performance was found between loaded and unloaded conditions (Table 3).

In a study by Bock et al. [15], the authors investigated the impact of movement patterns on marksmanship, as assessed by the Functional Movement Screen (FMS) tool. The subjects who passed the shooting assessment, based on their shooting score, were compared to those who failed, and no difference was found in the FMS scores between the two groups [15].

### 3.3. Psychological Factors

Eight studies [3,19,24,25,27,28,29,32] investigated the correlation of psychological factors and shooting performance (Table 3). Of these studies, six [19,24,25,27,28,32] investigated marksmanship under two different conditions: low-threat (LT) and high-threat (HT; Supplementary Table S2). In the LT condition, sometimes referred to as low-anxiety or low-pressure, the subjects either shot against a static target (mannequin) or against an experienced firearms instructor who did not shoot back. In the HT scenario, also referred to as high-anxiety or high-pressure, subjects shot against an experienced firearms instructor who shot back, causing some degree of pain in the participants. Colin et al. [19] assessed the effect of imagery on marksmanship in a HT condition. The researchers assigned the participants into three groups, with participants shooting in both LT and HT conditions (Supplementary Table S2) [19]. One group imagined successful shot execution under HT (EI group), another group imagined successful shot execution in HT including the accompanying emotions (EEI group), or a control group (CG) who had no imagery intervention [19]. No intervention was conducted in the LT condition. The authors reported that there was no significant difference between shooting performance in LT and HT for EI and EEI groups [19]. In contrast, the CG significantly decreased shot accuracy from LT to HT [19].

In two similar studies, Landman et al. [24,25] compared shooting performance in LT and HT conditions. In one of the studies [25], the authors reported significant positive correlations between HT marksmanship and decision-related action orientation (AOD, assessed via questionnaire) and sex (male); however, no correlation was found between shot accuracy and the State-Trait Anxiety Inventory (STAI), age, and work experience. Additionally, the authors reported the HT shot accuracy was predicted by AOD [25]. In the second study [24], the authors found significant positive correlations between HT shot accuracy and Thrill- and Adventure-Seeking (TAS, a subscale of the Sensation Seeking Scale), experience as a specialist officer, and previous exposure to violence when working as a police officer. The authors also reported that HT marksmanship was predicted by LT shot accuracy, TAS, and the Behavioral Inhibition System scale [24]. 

Nieuwenhuys and Oudejans conducted two studies assessing the impact of HT conditions on marksmanship [27,28]. In the first study [27], the researchers reported that shooting performance significantly declined in HT. In a further investigation [28], the researchers assessed the effects of training under HT on HT shooting performance. In this study, the participants were assigned to two groups that were exposed to four weekly training sessions of one hour [28]. The only difference between the groups was that the experimental group (EG) practiced under HT, whereas the CG practiced under LT. Two assessments were conducted, one two weeks after the last training session (post-test) and another four weeks after the last training session (retention test). At the post-test, the EG performed significantly better than the CG when shooting in HT [28]. However, no differences between the groups were found in the retention test, and the authors suggested that the significant improvement of the CG performance in HT may explain this finding [28]. In a similar intervention, Oudejans [32] assessed the effect of three weekly training sessions of 1 h between pre-test and post-test, exposing both EG and CG to the same training procedure, the only difference between the groups being the exposure of the EG to HT during the sessions. The author found a significant deterioration of marksmanship from LT to HT for both groups in the pre-test; however, in the post-test, only the CG significantly decreased performance from LT to HT, whereas the EG did not [32].

Of the remaining two studies, Kayihan et al. [3] reported a statistically significant negative correlation between state anxiety (assessed by STAI) and pistol shooting, while Oron-Gilad et al. [29] failed to find a correlation between marksmanship accuracy and workload. The authors of the latter study [29] used a subjective workload assessment (raw NASA task load index scores) and concluded that a higher perceived workload does not necessarily correlate to poor shooting performance or vice versa.

### 3.4. Other Factors

The study by Anderson and Plecas [5] also compared marksmanship results between two different handguns, namely Beretta model 94F and Glock model 22. It was reported that the type of gun significantly impacted shooting results, with participants using the Glock achieving significantly higher scores [5].

Finally, Copay and Charles [20] investigated the shooting performance of police recruits in four different low-light conditions with or without night sights. It was reported that night sights were correlated with significantly higher scores in all four low-light shooting conditions [20].

## 4. Discussion

The aim of this review was to identify and synthesize peer-reviewed published literature reporting on factors affecting marksmanship in law enforcement personnel. The review included 23 studies of different study designs, reporting a variety of factors and their correlations with shooting performance.

### 4.1. Grip Strength

Seven studies [3,5,7,16,21,26,31] included in this review assessed the association between marksmanship accuracy and grip strength, revealing mixed results. While the investigations by Anderson and Plecas [5], Brown et al. [16], Copay and Charles [21], Kayihan et al. [3], and Orr et al. [7] all reported a statistically significant positive correlation between shooting performance and hand grip strength, the studies by Muirhead et al. [26] and Orr et al. [31] failed to confirm this association. Moreover, Muirhead et al. [26] found a statistically significant negative correlation between grip strength and marksmanship accuracy when assessing shooting performance in a dynamic environment. Similarly, Orr et al. [31] reported a trend toward decreased performance in male officers as their grip strength increased; however, this association was not statistically significant.

It is possible that the type of handgun (trigger pull weight) plays a role in the association between grip strength and shooting accuracy, as suggested by Brown et al. [16]. For instance, Anderson and Plecas [5] found that the shooting performance of officers using Glock 22 (trigger pull weight of approximately 2.5 kg (5.5 lbs)) was better than those using Beretta 94F (trigger pull weight of approximately 5.0 kg (11 lbs)). However, this association needs to be interpreted with caution, as the results of two other included studies investigating officers using guns with comparable trigger pull weights (approximately 4.5 kg (10 lbs); Supplementary Table S2) reported conflicting results, with Brown et al. [16] finding a positive association between grip strength and marksmanship accuracy, while in the study by Muirhead et al. [26], this correlation was negative (Table 3).

Previous studies have suggested that an imbalance in the vascular supply to the distal arm may occur when the hand grip is not strong enough to handle the trigger weight [16,34,35]. As a consequence, the fine motor skills of the shooter deteriorate, and it may also affect the capacity to focus on the target and to deal with recoil. The importance of grip strength is further highlighted by the results reported in research conducted by Rodd et al. [36]. In their study, grip strength explained approximately 7% of the variation in marksmanship accuracy [36], a finding replicated in the included research by Kayihan et al. [3]. Additionally, when comparing hand grip strength between men and women, previous studies have demonstrated that, on average, male individuals have greater strength than females [37,38]. This difference in grip strength between sexes may explain the differences in shooting scores observed between male and female officers, as suggested by Orr et al. [31] and Brown et al. [16]. Brown et al. [16] were also able to estimate the minimum grip strength required to pass PPQ assessment, reported as 80 lbs. (approximately 36 kg). Furthermore, based on the results of two included studies [21,31], the positive correlation between shooting performance and grip strength may reach a certain threshold, above which the association may disappear. Of note, the included study by Copay and Charles [21] found that officers who trained grip strength using a hand-held device for eight weeks (Supplementary Table S2) had similar shooting performance to those officers who did not train grip strength (Table 3). However, the authors discussed that it was not possible to determine the level of adherence to the training intervention [21].

### 4.2. Physical Exertion

Physical exertion may not affect marksmanship (shooting accuracy, time, and precision) in practical scenarios. This finding is based on two included studies [17,22] and is supported by previous research [6,39,40].These previous investigations both reported that physical exertion did affect the rate of perceived exertion, blood lactate, mean HR during shooting, and time to complete the allocated tasks; however, shooting performance and scores remained unchanged [6,39,40]. Interestingly, the included study by Billich et al. [2] reported that the average distance between the hits on the target significantly increased after physical effort. However, the distance to the target in their study [2] was slightly higher than the previous investigations, with subjects shooting at 15 m from the target, whilst this distance was below 10 m in the other studies [16,22]. This finding suggests that potential fatigue effects may become more impactful at greater distances. However, it must be acknowledged that most shooting occurrences in real scenarios for law enforcement officer happen within five meters [41,42]. Additionally, Billich et al. [2] reported that the worst shooting performance found in their study was accurate enough to hit the target, and the values of dispersion of the shots from the central point were not statistically different from those measured at rest.

### 4.3. Tactical Load

Results of our review support the idea that tactical loads (i.e., the weight of equipment worn by officers) do not negatively affect shooting performance, based on three trials assessing specialist unit police officers [18,30,33]. This finding contrasts with what is reported by studies assessing shooting performance in military personnel [43,44]. However, the military occupational load is known to be heavier than the load carried by police officers [30,33], and, as suggested by Carlton et al. [45], loads weighing <25% of the subjects’ body mass may not affect performance. Interestingly, in the study by Orr et al. [30], the officers reported a subjective perception that their marksmanship accuracy (rifle and sidearm) was better when equipped with the usual occupational load, potentially a consequence of training under loaded conditions.

### 4.4. High-Threat Scenarios

The correlation between exposure to an HT scenario and shooting performance was investigated by six studies included in this review [19,24,25,27,28,32], with all studies finding that the stress related to an HT situation significantly increased anxiety levels in the officers. It is important to note that a significant negative correlation between anxiety and marksmanship was found in the study by Kayihan et al. [3]. Additionally, Nieuwenhuys and Oudejans [27] found that officers performed significantly worse in HT when compared to LT. As such, the evidence suggests that the anxiety imparted by HT scenarios may negatively impact marksmanship. However, some interventions can positively impact marksmanship under threat. Imagining the HT scenario and associated successful shot execution was reported by Colin et al. [19] as an effective method to mitigate performance deterioration under stress. Additionally, in two other studies [28,32], training under pressure significantly improved shooting performance. The investigation conducted by Oudejans [32] exposed the officers to three weekly sessions of 1 h, and the author reported that, when shooting in HT, subjects exposed to HT training were able to maintain the same shooting performance they had at LT, which was not the case for the CG. A similar intervention by Nieuwenhuys and Oudejans [28] revealed that the EG, exposed to training under stress, had significantly better shooting performance in the HT scenario than the CG. However, this result was not seen in the test conducted four weeks after the last training session, which suggests that continuing training may be necessary to maintain the positive results.

Landman et al. [24,25] reported factors that significantly correlate with marksmanship under HT in two separate studies. One of the studies [25] highlighted that the AOD questionnaire (related to the capacity to exert self-control) was not only positively correlated with, but also a strong predictor of, shooting performance. In the second study [24], experience, previous exposure to violence while on duty, and a high score on the TAS questionnaire (meaning affinity with risky activities) were reported as positively correlated to shooting performance under stress. The authors also noted that the TAS questionnaire could predict HT marksmanship [24].

### 4.5. Other Factors

Correlations were reported between marksmanship and the following factors: subjective workload perception (no significant correlation) [29], night sights (night sights significantly improved shooting performance in low-light conditions) [20], movement patterns (assessed by FMS, no significant correlation) [15], sex (males performed significantly better than females) [25], coordination (significant positive correlation) [3], flexibility (significant positive correlation) [3], balance (significant positive correlation) [3], and subjective workload perception (no significant correlation) [29]. However, these factors were investigated by single studies, and, therefore, findings should be interpreted with caution.

### 4.6. Limitations

Although this review was based on a thorough search of the literature in seven databases and included a significant number of studies from a variety of countries (including one investigation in Portuguese, which was translated to English), limitations should be acknowledged. Of note, while the search term development and extracted data were confirmed by multiple authors, the screening process and data extraction were conducted by a single author. Additionally, there was high heterogeneity among the included studies. For instance, they varied in design, population assessed (general duties Police Officers versus specialist unit Police Officers), age of participants, years of experience in service, shooting settings, courses of fire, distances to the target, types of handguns, and stances when shooting. Additionally, the difference in study designs makes it difficult to interpret the impact of confounding factors. Furthermore, the variability in marksmanship assessment (accuracy/performance) was also problematic. Finally, the lack of quality assessment of the included studies should also be highlighted, as it may erroneously suggest that all included studies are of the same quality and scientific rigor. Therefore, the results of this review, notably the findings of single individual studies, should be interpreted with caution.

## 5. Conclusions

The research synthesized in this review suggests that physical exertion does not decrease shooting performance, nor does tactical load carriage (up to circa 22 kg), with the latter subjectively reported by officers as improving performance. However, the physical capability of officers may be of importance, notably grip strength, which the volume of evidence suggests is positively correlated with marksmanship up to a given point. Additionally, although anxiety was found to negatively impact shooting performance, studies indicate that training under stress may counteract this factor, albeit for a short period (approximately four weeks), highlighting that training needs to be continued to maintain the positive results. Together, all these aforementioned factors appear to have a trainability component, where increased specific and realistic training, can improve shooting accuracy, time, and precision, especially under high-stress situations.

## Figures and Tables

**Table 1:** Eligibility criteria for identified studies.

Inclusion Criteria	Exclusion Criteria
Peer-reviewed publications reporting original research.	Studies that only reported results for weapons other than handguns, or results for handgun were not separately reported.
English availability or translatable to English.	Studies assessing marksmanship via virtual reality, simulators, or in competitions.
Subjects were police officers or police recruits.	
Objective assessment of marksmanship.	
Reported handgun shooting accuracy.	
Investigated the impact of at least one factor on marksmanship.	

**Table 2:** Search terms employed to search the PubMed database.

Police		Marksmanship
(“police”[MeSH Terms] OR “police”[All Fields] OR “polices”[All Fields] OR “policing”[All Fields] OR “law enforcement”[All Fields] OR “tactic”[All Fields] OR “tactical”[All Fields] OR “tactically”[All Fields] OR “tactics”[All Fields] OR “sheriff”[All Fields] OR “sheriffs”[All Fields])	**AND**	(“marksmanship”[All Fields] OR ((“perform”[All Fields] OR “performance”[All Fields] OR “performances”[All Fields] OR “accuracies”[All Fields] OR “accuracy”[All Fields] OR “precise”[All Fields] OR “preciseness”[All Fields] OR “precision”[All Fields] OR “precisions”[All Fields] OR “efficiency”[MeSH Terms] OR “efficiency”[All Fields] OR “efficient”[All Fields] OR “efficiently”[All Fields] OR “lethal”[All Fields] OR “lethalities”[All Fields] OR “lethality”[All Fields] OR “lethals”[All Fields] OR “handle”[All Fields] OR “handled”[All Fields] OR “handles”[All Fields] OR “handling”[All Fields] OR “han-dlings”[All Fields] OR “skill*”[All Fields]) AND (“handgun *”[All Fields] OR “weapon *”[All Fields] OR “gun”[All Fields] OR “shoot *”[All Fields] OR “firing”[All Fields] OR “firings”[All Fields] OR “pistol *”[All Fields] OR “rifle *”[All Fields] OR “shot”[All Fields] OR “firearm *”[All Fields])))

**Table 3:** Data extraction table detailing the population, variables, and research results of included studies.

Reference /Study Design	Population	Variables	Results
Anderson and Plecas 2000 [5] Cross-sectional design	* Police Recruits (n = 65; 54 males) * Mean age: NR * Mean experience: NR	* 30-trigger pull test * Balance * Forearm girth * Grip strength * Hand breadth * Hand length * Handgun type: # Beretta 94F # Glock 22 * Height * Humerus length * POPAT * Sex * Static push-up * Thumb length * Trigger finger length * Ulnar length * Weight * Wrist breadth	* When males and females were analyzed together, shooting scores were significantly correlated with: # Forearm girth # Dominant grip strength # Combined grip strength # Second ray length # POPAT times * No significant relationships were found in the sex-specific groups * Participants using Beretta had significantly lower qualifying scores than those using Glock * No other correlations were found
Billich et al. 2014 [2] Quasi-experimental design	* Specialist Police Officers (n = 8) * Mean age: 30.3 (±3.5) y	* Physical exertion	* The distance between hits significantly increased after physical exertion * Physical exertion negatively affected shooting precision
Bock et al. 2016 [15] Cross-sectional design	* Police Recruits (n = 53) * Minimum age: 18 y and 4 months * Mean age: NR * Mean experience: NR	* FMS score	* FMS scores were similar when comparing those passing to those failing the marksmanship assessment
Brown et al. 2021 [16] Cross-sectional design	* Police Officers (n = 118; 86 males): # Constables: 79.6% # Corporals: 14.2% # Sergeants: 5.3% # Staff Sergeants: 0.9% * Mean age: 36.2 (±7.8) y	* Grip strength (dominant hand) * Sex	* Grip strength was significantly positively correlated with PPQ scores (only when sexes were analyzed together) * Grip strength values in the range of 36.3kg and 56.7kg (80 lb and 125 lb) were needed to score approximately 85% and 90% on the PPQ (passing scores), respectively * Female officers achieved lower PPQ scores and had lower grip strength compared to their male counterparts
Brown et al. 2013 [17] Quasi-experimental design	* Police Officers (n = 8) * Mean age: 30.1 (±5.9) y * Mean experience: 4.4 (±2.0) y	* HR * Physical exertion (85% HR_max_ or exhaustion)	* Neither HR nor physical exertion were correlated with shooting performance * Similar shooting performance between pre- and post-exercise
Carbone et al. 2014 [18] Crossover trial	* Specialist Police Officers (n = 6) * Mean age: 33.3 (±4.1) y * Mean experience Police Officer: 10.9 (±5.1) y * Mean experience Specialist Police Officer: 4.0 (±2.8) y * Minimum fitness standard	* Shooting trials: # Static trial # Mobile trial * Tactical load (mean weight 22.8 ± 1.8 kg)	* In both trials, DCOT and vertical dispersion (Y) were similar when comparing loaded and unloaded conditions * In the static trial, the horizontal shot dispersion (X) significantly improved when tactically loaded; however, no difference was found in the mobile trial
Colin et al. 2014 [19] Randomized controlled trial	* Police Officers (n = 66; 53 males) * Mean age: 29.7 (±9.1) y * Mean experience: 6.1 (±7.2) y	* Shooting conditions: # LT: opponent armed with a dummy handgun (Bluegun^®^, Walther P5; no trigger) # HT: opponent armed with a real handgun (Walther P5), with the opponent occasionally shooting back using colored-soap cartridges	* The CG performed significantly worse when shooting in the HT condition, compared to the performance in LT * No difference in shooting accuracy was found between LT and HT conditions for subjects in the execution imagery and execution-emotion imagery groups * Both execution imagery and execution-emotion imagery groups performed significantly better than the CG when shooting in the HT condition
Copay and Charles 2001 [20] Cross-sectional design	* Police Recruits (n = 149): # Less skilled shooters (n = 65) # Skilled shooters (n = 79) * Mean age: NR * Mean experience: NR	* Night shooting conditions: # Back-lighted target # Front-lighted target # With flashlight (three different types) # Intermittent lights * Shooting skill * Use of night sights	* Skilled shooters performed significantly better than less skilled shooters when firing in the back-lighted, front-lighted, and flashlight conditions, regardless of night sight use. No difference was found between shooting skills in the intermittent light condition * For both less skilled and skilled shooters, shooting scores using flashlights were significantly lower than the scores in the front-lighted target condition * Type of flashlight used did not affect accuracy * For both less skilled and skilled shooters, the shooting scores in all four shooting conditions were significantly higher for officers who used night sights compared to those that did not
Copay and Charles 2001 [21] Quasi-experimental design	* Police Recruits # CG: n = 91 (81 males) # EG: n = 96 (79 males) * Mean age: NR * Mean experience: NR	* Grip strength (dominant hand) * Sex	* Shooting scores significantly improved for both CG and EG after 8 weeks of training, with no significant differences between the groups * Grip strength was positively correlated with marksmanship performance and was a predictor of the shooting scores in the post-training assessment * In both pre- and post-training assessments, female recruits had lower grip strength and marksmanship scores than their male counterparts * Sex was not a predictor of shooting performance when grip strength was accounted for
Do Nascimento Neto et al. 2017 [22] Quasi-experimental design	* Specialist Police Officers (n = 15) * Mean age: 34.1 (±5.4) y * Mean experience ≥ 6 y	* Aerobic capacity (VO_2 peak_) * Physical exertion (85% HR_max_)	* VO_2 peak_ was not correlated with marksmanship performance when shooting at rest and after the exercise intervention * Physical exertion did not affect shooting performance
Hornsby et al. 2021 [23] Quasi-experimental design	* Police Officers, n = 10 (8 males) * Mean age: 32.2 (±9.4) y	* HR biofeedback	* The use of HR biofeedback did not improve marksmanship accuracy
Kayihan et al. 2013 [3] Cross-sectional design	* Police Recruits (n = 237) * Age range: 19-20 y	* Aerobic capacity (20-MST) * Age * Anxiety (state anxiety, trait anxiety, change in anxiety) * Balance (Stork Balance Standing Test) * Biceps circumference * BMI * Calf circumference * Coordination (Alternate Hand Wall Toss Test) * Femur diameter * Flexibility (sit and reach test) * Grip strength (dominant hand) * Height * HR * Humerus diameter * Muscular endurance (curl-up test: 30 and 60 sec) * Reaction time * Skinfold thickness (abdominal, pectoral, thigh) * Weight * Wrist circumference	* Significant correlations were found between shooting efficiency and: # Anxiety variability (negative correlation) and state anxiety (negative correlation) # Balance (positive correlation) # Biceps circumference (positive correlation) # Coordination (positive correlation) # Femur diameter (positive correlation) # Flexibility (positive correlation) # Grip strength (positive correlation) # HR variability during shooting (negative correlation) # HR_max_ (negative correlation) # Mean HR (negative correlation) # Wrist circumference (positive correlation) * No other correlations were found
Landman et al. 2016 [24] Cross-sectional design	* Police Officers (regular officers; n= 14) # Mean age: 34.6 (±9.4) y * Specialist Police Officers (AU officers; n = 15) # Mean age: 29.4 (±3.1) y * Officers enrolled to start Specialist Police (pre-AU officers; n = 11) # Mean age: 30.6 (±5.0) y	* Anxiety (STAI) * BIS * Experience (work experience, experience with violent situations, army experience) * FI * Self-control strength (AOD, AOT) * Shooting conditions: # LP: opponent fired a blank cartridge # HP: opponent fired blank and colored-soap cartridges * TAS	* AU officers shot significantly more accurately than the regular officers * Officers (combined) shot more accurately in the LP condition than in the HP condition * HP shooting accuracy was positively correlated with: # AU experience # TAS # Violence experience * HP shooting accuracy was predicted by: # AU experience # BIS # LP shooting accuracy # Order of conditions (LP x HP) # TAS * No other correlations were found
Landman et al. 2016 [25] Cross-sectional design	* Police Officers (n = 42; 37 males) * Mean age: 32.9 (±8.3) y * Mean experience: 9.0 (±7.6) y	* Anxiety (STAI) * Age * Experience (work experience) * Self-control strength (AOD, AOT) * Sex * Shooting conditions: # LP: the opponent was a life-size mannequin dressed in a black protective overall, facemask, throat protector, and hand gloves # HP: the opponent was an experienced police firearms instructor wearing the same clothes and protective gear as the mannequin in LP, occasionally firing colored soap cartridges	* HP shooting accuracy was positively correlated with: # AOD # Sex (male) * HP shooting accuracy was predicted by: # AOD # LP shooting accuracy * No other correlations were found
Muirhead et al. 2019 [26] Retrospective cohort	* Police Officers (n = 33) * Mean age: 40.5 (±6.7) y	* Aerobic capacity (20-MST) * Grip strength (dominant hand) * Isometric leg/back strength * Shooting scenarios: # Static # Dynamic # Positive identification * Vertical jump	* Static scenario: # Shooting accuracy was positively correlated with 20-MST * Dynamic scenario: # Shooting accuracy was negatively correlated with dominant grip strength * Positive identification scenario: # Shooting accuracy was positively correlated with leg/back strength * No other correlations were found
Nieuwenhuys and Oudejans 2010 [27] Cross-sectional design	* Police Officers (n = 7; 6 males) * Mean age: 23.8 (±2.0) y * Mean experience: 3.4 (±2.4) y	* Shooting conditions: # LA: the opponent was a life-size mannequin dressed in a black protective overall, facemask, throat protector, and hand gloves # HA: the opponent was an experienced police firearms instructor wearing the same clothes and protective gear as the mannequin in LP, occasionally firing colored soap cartridges	* Shooting performance was significantly lower under HA compared to LA
Nieuwenhuys and Oudejans 2011 [28] Randomized controlled trial	* Police Officers (n = 27; 25 males) # CG n = 14 (13 males); mean age: 34.8 (±6.4) y; mean experience: 11.5 (±5.7) y # EG: n = 13 (12 males); mean age: 34.6 (±7.4) y; mean experience: 11.6 (±7.0) y	* Shooting conditions: # LA: the opponent was a life-size mannequin dressed in a black protective overall, facemask, throat protector, and hand gloves # HA: the opponent was an experienced police firearms instructor wearing the same clothes and protective gear as the mannequin in LP, occasionally firing colored soap cartridges	* Pre-test: # Both EG and CG performed significantly worse under HA compared to LA * Post-test: # Shooting performance in the CG significantly decreased from LA to HA # Shooting performance in the EG was similar between HA and LA # The EG performed significantly better than the CG when shooting under HA * Retention test (at 4 months): # Shooting performance in the CG significantly decreased from LA to HA # Shooting performance in the EG was similar between HA and LA # The CG performed significantly better shooting under HA compared to shooting under HA at the post-test
Oron-Gilad et al. 2008 [29] Cross-sectional design	* Police Officers (n = 62; 52 males) * Mean age: 37.0 (range 22–56) y * Mean experience: 11.0 (range 1–32) y	* Age * Experience (years of service) * Night shooting tasks: # Warmup task # Flashlight task # Barrel task # Metal task * Subjective workload (RTLX scores)	* Age and years of service were not correlated with shooting performance * RTLX scores significantly increased and shooting performance significantly decreased from: # Warmup task to flashlight task # Warmup task to barrel task # Flashlight task to barrel task * Performance was similar comparing shooting in the flashlight task and in the metal task, despite a significant increase in RTLX scores (higher in the latter) * Performance in the warmup task was predictive of their performance in the other tasks * Performance in the warmup, flashlight, and barrel tasks were correlated with each other * Performance in the metal task did not correlate with any of the other tasks * In the metal task, shooting performance was negatively correlated with task duration
Orr et al. 2018 [30] Crossover trial	* Specialist Police Officers (n = 6) * Mean age: 34.0 (±7.4) y * Mean experience: 6.0 (±6.8) y	* Tactical load (mean weight 23.5 kg ± 2.8 kg) * Shooting trials: # Short forward movement # Mobility task * Subjective perception of the impact of load carriage on performance	* Marksmanship accuracy was similar when comparing performance under loaded and unloaded conditions in both shooting trials * Officers perceived improved marksmanship when tactically loaded * No other correlations were found
Orr et al. 2017 [7] Retrospective cohort	* Police Recruits (n = 169) * Minimum age: 18 y and 4 months	* Grip strength	* Grip strength (both right and left sides) predicted marksmanship score * Individuals who passed the marksmanship assessment had significant higher grip strength (both right and left sides) than those who failed
Orr et al. 2021 [31] Cross-sectional design	* Police Officers (n = 12; 6 males) * Mean age: 38.1 (±6.2) y * Mean height: 174.4 (±7.3) cm	* Grip strength (dominant hand) * Hand size (dominant hand): # Palm width across the MCP joints # Hand span (measured from the tip of the pollex to the tip of the 5th phalange) * Sex	* Neither hand size nor grip strength were correlated with marksmanship performance * Non-significant trend towards better DCOT scores for: # Female officers with higher grip strength # Female officers with larger hand span * Non-significant trend towards worse DCOT scores for: # Male officers with higher grip strength # Male officers with larger hand span
Oudejans 2008 [32] Quasi-experimental design	* Police Officers (n = 17; 15 males) # CG (n = 8; 7 males); mean age: 39.0 (±8.5) y; mean experience: 14.0 (±8.0) y # EG (n = 9; 8 males); mean age: 35.0 (±9.5) y; mean experience: 9.0 (±5.4) y	* Shooting scenarios: # LP: shooting at cardboard targets # HP: shooting against a certified firearms instructor dressed in a black protective overall, facemask, throat protector, and hand gloves, carrying a fake knife and a handgun, occasionally firing marking cartridges	* Pre-training: # Shooting performance was worse under HP compared to shooting under LP for both EG and CG * Post-training: # EG had similar performance when shooting under HP and LP # CG had lower shooting performance under HP compared to LP
Thomas et al. 2018 [33] Quasi-experimental design	* Specialist Police Officers (n = 12) * Mean age: 33.7 (±5.2) y * Mean experience in law enforcement: 8.8 (±4.4) y * Mean experience in SWAT: 4.8 (±4.6) y	* Tactical load (mean weight 14.2 ± 2.0 kg)	* No differences when shooting under loaded and unloaded conditions during the STT

*Abbreviations:* 20-MST: 20-Meter Shuttle Run Test; AOD, Decision Related Action Orientation; AOT, Failure or Threat Related Action Orientation; AU, Arrest Unit; BIS, Behavioral Inhibition Scale; cm, centimeters; CG, Control Group; DCOT, Distance to Centre of Target; EG, Experimental Group; FI, Functional Impulsivity; FMS, Functional Movement Screen; HA, High Anxiety; HP, High Pressure; HR, Heart Rate; HR_max_, Maximum Heart Rate; HT, High Threat; kg, kilogram; LA, Low Anxiety; lb, pound; LP, Low Pressure; LT, Low Threat; MCP, Metacarpophalangeal; NR, Not Reported; POPAT, Police Officer Physical Abilities Test; PPQ, Police Pistol Qualification; RTLX, Raw NASA Task Load Index; sec, seconds; STAI, State-Trait Anxiety Inventory; STT, Simulated Tactical Test; SWAT, Special Weapons and Tactics Operators; TAS, Thrill and Adventure Seeking scale; VO_2 peak_, Peak Oxygen Uptake; y, years.

## Data Availability

Not applicable.

## Supplementary Materials

The following supporting information can be downloaded at: https://www.mdpi.com/article/10.3390/ijerph192114236/s1, Table S1: Search terms employed to search according to database; Table S2: Data extraction table detailing countries, interventions, and marksmanship assessments.
