# Supplementary material for: Factors Influencing Marksmanship in Police Officers: A Narrative Review
Source: Int J Environ Res Public Health. 2022 Oct 31;19(21):14236. doi: 10.3390/ijerph192114236 (PMC9655518; doi:10.3390/ijerph192114236)
Supplement: Supplementary file 1 [file ijerph-19-14236-s001.zip › ijerph-1926741-Table S1.pdf]

Supplementary Table S1. Search terms employed to search according to database.

Database	Concepts		
	Police		Marksmanship
CINAHL	(((((((MH "police+") OR police) OR polices) OR policing) OR "law enforcement") OR (((tactic OR tactical) OR tactically) OR tactics)) OR (sheriff OR sheriffs))	**AND**	(marksmanship OR (((((((((perform OR performance) OR performances) OR (accuracies OR accuracy)) OR (((precise OR preciseness) OR precision) OR precisions)) OR (((((MH "efficiency+")) OR efficiency) OR efficient) OR efficiently)) OR (((lethal OR lethalities) OR lethality) OR lethals)) OR ((((handle OR handled) OR handles) OR handling) OR handlings)) OR skill*) AND ((((((((handgun* OR weapon*) OR gun) OR rifle*) OR shoot*) OR ((firing) OR firings)) OR pistol*) OR shot) OR firearm*)))
Cochrane	((((((([mh police] OR police) OR polices) OR policing) OR "law enforcement") OR (((tactic OR tactical) OR tactically) OR tactics)) OR (sheriff OR sheriffs))	**AND**	(marksmanship OR (((((((((perform OR performance) OR performances) OR (accuracies OR accuracy)) OR (((precise OR preciseness) OR precision) OR precisions)) OR (((([mh efficiency]) OR efficiency) OR efficient) OR efficiently)) OR (((lethal OR lethalities) OR lethality) OR lethals)) OR ((((handle OR handled) OR handles) OR handling) OR handlings)) OR skill*) AND ((((((((handgun* OR weapon*) OR gun) OR rifle*) OR shoot*) OR ((firing) OR firings)) OR pistol*) OR shot) OR firearm*)))
Embase	(((((('police'/exp OR police) OR polices) OR policing) OR "law enforcement") OR (((tactic OR tactical) OR tactically) OR tactics)) OR (sheriff OR sheriffs))	**AND**	(marksmanship OR (((((((((perform OR performance) OR performances) OR (accuracies OR accuracy)) OR (((precise OR preciseness) OR precision) OR precisions)) OR (((('efficiency'/exp) OR efficiency) OR efficient) OR efficiently)) OR (((lethal OR lethalities) OR lethality) OR lethals)) OR ((((handle OR handled) OR handles) OR handling) OR handlings)) OR skill*) AND ((((((((handgun* OR weapon*) OR gun) OR rifle*) OR shoot*) OR ((firing) OR firings)) OR pistol*) OR shot) OR firearm*)))
Scopus	((((((INDEXTERMS("police") OR "police") OR "polices") OR "policing") OR "law enforcement") OR ((("tactic" OR "tactical") OR "tactically") OR "tactics")) OR ("sheriff" OR "sheriffs"))	**AND**	("marksmanship" OR ((((((((("perform" OR "performance") OR "performances") OR ("accuracies" OR "accuracy")) OR ((("precise" OR "preciseness") OR "precision") OR "precisions")) OR ((((INDEXTERMS("efficiency")) OR "efficiency") OR "efficient") OR "efficiently")) OR ((("lethal" OR "lethalities") OR "lethality") OR "lethals")) OR (((("handle" OR "handled") OR "handles") OR "handling") OR "handlings")) OR "skill*") AND

			(((((((("handgun*" OR "weapon*") OR "gun") OR "rifle*") OR "shoot*") OR (("firing") OR "firings")) OR "pistol*") OR "shot") OR "firearm*")))
SPORTDiscus	(((((((MH "police+") OR police) OR polices) OR policing) OR "law enforcement") OR (((tactic OR tactical) OR tactically) OR tactics)) OR (sheriff OR sheriffs))	**AND**	(marksmanship OR (((((((((perform OR performance) OR performances) OR (accuracies OR accuracy)) OR (((precise OR preciseness) OR precision) OR precisions)) OR (((((MH "efficiency+")) OR efficiency) OR efficient) OR efficiently)) OR (((lethal OR lethalities) OR lethality) OR lethals)) OR ((((handle OR handled) OR handles) OR handling) OR handlings)) OR skill*) AND ((((((((handgun* OR weapon*) OR gun) OR rifle*) OR shoot*) OR ((firing) OR firings)) OR pistol*) OR shot) OR firearm*)))
Web of Science	((((((police OR police) OR polices) OR policing) OR "law enforcement") OR (((tactic OR tactical) OR tactically) OR tactics)) OR (sheriff OR sheriffs))	**AND**	(marksmanship OR (((((((((perform OR performance) OR performances) OR (accuracies OR accuracy)) OR (((precise OR preciseness) OR precision) OR precisions)) OR ((((efficiency) OR efficiency) OR efficient) OR efficiently)) OR (((lethal OR lethalities) OR lethality) OR lethals)) OR ((((handle OR handled) OR handles) OR handling) OR handlings)) OR skill*) AND ((((((((handgun* OR weapon*) OR gun) OR rifle*) OR shoot*) OR ((firing) OR firings)) OR pistol*) OR shot) OR firearm*)))
