# Supplementary material for: Factors Influencing Marksmanship in Police Officers: A Narrative Review
Source: Int J Environ Res Public Health. 2022 Oct 31;19(21):14236. doi: 10.3390/ijerph192114236 (PMC9655518; doi:10.3390/ijerph192114236)
Supplement: Supplementary file 1 [file ijerph-19-14236-s001.zip › ijerph-1926741-Table S2.pdf]

Supplementary Table S2. Data extraction table detailing countries, interventions, and marksmanship assessments.

REFERENCE	COUNTRY	INTERVENTION	MARKSMANSHIP ASSESSMENT
Anderson & Plecas 2000 [5]<br><br>Predicting shooting scores from physical performance data	Canada	N/A	* Marksmanship course: BC48<br>* Target: B27<br>* Distance to target: NR<br>* Handguns: Beretta 94F and Glock 22<br>* Accuracy/performance: five points for each of the 48 shots (total of 240 points; passing score: 192); shooting scores: final scores recorded after 4 days of shooting
Billich et al. 2014 [2]<br><br>Effect of maximum heart rate on accuracy of fire	Czech Republic	* Shooting at normal resting HR<br>* Physical exertion: exercise on a treadmill, 2 min warm-up at 5.5km/h, then speed increased up to 10km/h, with intensity continuously increasing by 2km/h every minute until participant refused to continue<br>* Shooting after physical exertion	* Marksmanship course: each subject used 10 bullets; the time limit for each shot fired was 2 sec; the total time for each round was 20 sec<br>* Target: standard shooting target No. 4<br>* Distance to target: 15 m<br>* Handgun: Glock 17<br>* Accuracy/performance: mean shot values analyzed based on the DCOT
Bock et al. 2016 [15]<br><br>The Functional Movement Screen as a predictor of police recruit occupational task performance	Australia	N/A	* Marksmanship course: 30 scoring rounds over several serials<br>* Target: standard police Z-4 target<br>* Distance to target: NR<br>* Handgun: Glock 9 mm<br>* Accuracy/performance: pass/fail assessment (passing score: 80 points)
Brown et al. 2021 [16]<br><br>Examining the impact of grip strength and officer gender on shooting performance	USA	N/A	* Marksmanship course: PPQ (five stages):<br># Stage 1: 14 rounds at a distance of 25m (82 feet) while standing (seven rounds), kneeling (five rounds), and in prone (two rounds).<br># Stage 2: eight rounds at a distance of 15m (49 feet) while standing (four rounds) and kneeling (four rounds). Participants had 20 sec to complete.<br># Stage 3: eight rounds at a distance of 7m (23 feet) away, two rounds while standing to the right side of their lane and two rounds while on the left side, repeating until shooting eight rounds.

REFERENCE	COUNTRY	INTERVENTION	MARKSMANSHIP ASSESSMENT
			# Stage 4: 12 rounds: three rounds (two aimed at centre mass and one aimed at the head) at 4.9m (16 feet) away for four, 5-sec intervals.<br># Stage 5: eight rounds (four with their dominant hand only and four with their support hand only) at a distance of 3m (10 feet). Participants had 15 sec to complete.<br>* Target: NR<br>* Distance to target: ranging from 3 to 25m (10 to 82 feet)<br>* Handgun: DAO pistol (trigger pull: 3.6 – 5.4kg [8 lbs-12 lbs])<br>* Accuracy/performance: pass/fail assessment (pass score: 200/250 – 80%)
Brown et al. 2013 [17]<br><br>The effect of acute exercise on pistol shooting performance of police officers	USA	* Shooting at rest<br>* Exercise: 4 min warm-up, then cycling (cycle ergometer) in 60 sec/30 sec work/rest intervals until reaching 85% HR_{max} or exhaustion<br>* Shooting within 15 sec of completing exercise	* Marksmanship course: 3 rounds of shooting separated by 15-sec rest period with subjects turning their back to the target; 55 rapid fire shots in each round (different shooting lane every round)<br>* Target: human silhouette<br>* Distance to target: 9.1m (10 yards)<br>* Handgun: duty pistols<br>* Accuracy/performance: average DCOT / group shot diameter (the diameter of the smallest circle that encompassed the tightest 4-shot grouping)
Carbone et al. 2014 [18]<br><br>The impact of load carriage on the marksmanship of the tactical police officer: a pilot study	Australia	* Addition of tactical load (mean weight 22.8 ± 1.8 kg)	* Marksmanship course:<br># Static trial: five single, deliberate, well aimed rounds from the standing unsupported firing position<br># Mobile trial: 25-m course, 10-m straight-line sprint followed by a tactical move through two doorways, a descent of seven stairs, move through another doorway and then target approach<br>* Target: 25 mm discoid target<br>* Distance to target: 6 m<br>* Handgun: Glock 9 mm<br>* Accuracy/performance: mean of the sum of DCOT / horizontal dispersion (X) / vertical dispersion (Y)
Colin et al. 2014 [19]<br><br>Positive effects of imagery on	The Netherlands	* Three intervention groups<br># EI: imagined successful shot execution. The imagery script	* Marksmanship course: 4 blocks (under LT and HT conditions). In each block, subjects fired 1 round at the opponent's left leg, 1 round at the right leg and 2 rounds at the chest. After finishing each block, participants

REFERENCE	COUNTRY	INTERVENTION	MARKSMANSHIP ASSESSMENT
police officers' shooting performance under threat		guided participants through the shooting exercise, pointing them specifically to successful shot execution<br># EEI: imagined successful shot execution under threat, including the accompanying emotions. The imagery script included the threat of being hit and effectively dealing with the associated stress and anxiety<br># CG: no imagery intervention	directly moved to a new position and continued with the next block, reloading their gun after the second block<br>* Target: opponent fitted with white target areas (one on the chest – 30×30 cm – and two on the upper legs – 12×35 cm). The opponent was an experienced firearms instructor, who stood straight up facing the participants, wearing a black protective overall, face mask, throat protector, and hand gloves<br>* Distance to target: 5 m<br>* Handgun: Walter P5 9 mm (duty weapon)<br>* Accuracy/performance: mean percentage of hits
Copay & Charles 2001 [20]<br><br>Handgun shooting accuracy in low light conditions. The impact of night sights	USA	N/A	* Marksmanship course: 3 night conditions:<br># Back-lighted target: source of light behind the target; 12 rounds at 13.7m (15 yards)<br># Front-lighted target: source of light in front of the targe; 12 rounds at 13.7m (15 yards)<br># With a flashlight: officers used three different flashlights to illuminate the target (Mag-Lite, Kel-Lite, Sure-Fire 6z); 12 rounds at 13.7m (15 yards)<br># Intermittent lights: intermittent source of light (flashing emergency lights); 14 rounds at 6.4m (7 yards)<br>* Target: yellow paper target, shape of the human upper body (down to below the waist) with 3 concentric rectangles drawn on the chest<br>* Distance to target: ranging from 6.4 to 13.7m (7 to 15 yards)<br>* Handgun: department issued weapon (wide range between subjects)<br>* Accuracy/performance: total score (10 points for hits in the center rectangle, 9 points for hits in the intermediate rectangle, 8 points for hits in the outer rectangle, and 1 point for hits on the target but outside the rectangles – maximum score: 140)

REFERENCE	COUNTRY	INTERVENTION	MARKSMANSHIP ASSESSMENT
Copay & Charles 2001 [21]<br><br>The influence of grip strength on handgun marksmanship in basic law enforcement training	USA	* EG: 8-weeks firearms training and grip strength training using a hand-held device ("Grip Master") with three levels of resistance: 2.3kg, 3.2kg, or 4.1kg (5, 7, or 9 lb) per finger. Subjects were free to choose their level of resistance during training. Male subjects trained with 4.1kg (9 lb) and female subjects chose either the 4.1 or 3.2kg (9 lb or 7 lb)<br>* CG: 8-weeks firearms training	* Marksmanship course: 14 rounds (pre- and post-intervention)<br>* Target: paper target, silhouette of a human upper body (down to below the waist) with three concentric rectangles drawn on the chest<br>* Distance to target: 13.7m (15 yards)<br>* Handgun: department-issued weapon<br>* Accuracy/performance: total score (10 points for the center rectangle, 9 points for the intermediate rectangle, 8 points for the outer rectangle, and 1 point for a shot on the target but outside the rectangles – maximum score: 140 points)
Do Nascimento Neto et al. 2017 [22]<br><br>Effect of physical effort on shooting performance of military officers of the riot police	Brazil	* Exercise intervention: 297-m obstacle course simulating real life scenarios, with participants achieving 85% HR_{max}	* Marksmanship course: 5 shots at the target in a standing position using both hands<br>* Target: human silhouette (height: 1.65 m) with a circular target at the center (center-of-mass)<br>* Distance to target: 8 m<br>* Handgun: Beeman 2004, 4.5 mm caliber, 0.77 kg<br>* Accuracy/performance: total score, shooting time, ratio between total score and shooting time
Hornsby et al. 2021 [23]<br><br>Effects of heart rate biofeedback, sleep, and alertness on marksmanship accuracy during a live-fire stress shoot	USA	* Groups:<br># EW (HR biofeedback) group: subjects placed their thumb on a HR monitor and were instructed to match their breathing rate with the lights on the device. Participants maintained this breathing pattern for 10 minutes before starting the stress shooting trial<br># PLA: subjects received two coconut flour capsules and rested for	* Marksmanship course: after a patrol car siren sounded and a loud music started playing, subjects ran 50 m to a set of stairs, climbed five stairs, entered a patrol vehicle, buckled the seat belt, and placed both hands on the steering wheel. After instruction, participants got out of the vehicle and approached a barricade 5 m away. At the barricade, participants shot 5 bullets at a target 19.2 m (21 yards). The same trial was repeated twice, with the participants shooting at the second barricaded (target 19.2 m [21 yards] away) and at the third barricade (target 13.7 m [15 yards] away)<br>* Target: steel target<br>* Distance to target: ranging from 13.7 m to 19.2 m (15 to 21 yards)<br>* Handgun: service weapon

REFERENCE	COUNTRY	INTERVENTION	MARKSMANSHIP ASSESSMENT
		10 minutes before starting the stress shooting trial<br>* Duration: two weeks	* Accuracy/performance: hit / no-hit
Kayihan et al. 2013 [3]<br><br>Relationship between efficiency of pistol shooting and selected physical-physiological parameters of police	Turkey	N/A	* Marksmanship course: 10 shots with one hand only from a standing position, with no support for the arm or wrist, within 20 min<br>* Target: ten-ring target<br>* Distance to target: 10 m<br>* Handgun: CZ 75B 9.0 mm (service pistol)<br>* Accuracy/performance: total score
Landman et al. 2016 [24]<br><br>The impact of personality traits and professional experience on police officers' shooting performance under pressure	The Netherlands	N/A	* Marksmanship course: 24 trials in each condition (LP and HP). After instruction, subjects faced the shooting range and aimed their gun. Around 3–5 sec later, an opponent appeared facing the officer. The opponent was armed in a randomized 50% of the trials (in 50% of the trials the opponent appeared from the left and in the other 50% he appeared from the right)<br># Armed trial: the opponent aimed a gun immediately upon appearance and participants were required to quickly fire 2 rounds<br># Unarmed trial: the opponent aimed a cartridge-clip similar to the gun and participants were required to refrain from shooting<br>* Target: white square (28×28 cm) fitted on the opponent's chest<br>* Distance to target: 5 m<br>* Handgun: 9mm duty weapon (Glock 17 for AU officers; Walther P5 or Walther P99Q for the regular and pre-AU officers)<br>* Accuracy/performance: number of hits in the armed trials, incorrect shooting decisions (number of shots in the unarmed trials)

REFERENCE	COUNTRY	INTERVENTION	MARKSMANSHIP ASSESSMENT
Landman et al. 2016 [25]<br><br>Decision-related action orientation predicts police officers' shooting performance under pressure	The Netherlands	N/A	* Marksmanship course: 10 trials in each condition (LP and HP). After instruction, officers fired 1 round at the opponent's right leg target, stepped to the right, fired 1 round at the opponent's left leg target, reloaded their handgun, fired 1 round at the opponent's chest target, stepped back to the left and fired one round at the chest target<br>* Target: opponent (either a life-size mannequin or a firearms instructor) fitted with white target areas (one on the chest: 28 cm x 28 cm, and two on the upper legs: 12 cm x 35 cm)<br>* Distance to target: 5 m<br>* Handgun: Walther P5 (service weapon)<br>* Accuracy/performance: mean percentage of hits for each condition

REFERENCE	COUNTRY	INTERVENTION	MARKSMANSHIP ASSESSMENT
Muirhead et al. 2019 [26]<br><br>The relationship between fitness and marksmanship in police officers	USA	N/A	* Marksmanship course: shooting in three dimly lit scenarios:<br># Static scenario: 10 rounds at own pace standing ten yards away from the target<br># Dynamic scenario: prior to shooting, officers performed a 3-min sub-maximal step-test. Then, officers shot two rounds 9.1m (10 yards) from the target behind "cover", then were instructed to "combat reload" and fire four rounds while moving closer to the target to the 1.4m-line (1.5-yard) . They then dragged a 99.8kg (220 lb) dummy backwards to the 9.1m-line (10-yard) and fired four rounds behind "cover"<br># Positive identification scenario: immediately after the dynamic scenario, the officers moved to the 9.1m-line (10-yard) and were given 4 sec to draw their guns, identify the targets and fire two rounds on "any backer with a number on it"<br>* Target: 20.3cm (8") circle located at the center-of-mass or on the head of a black silhouette in the shape of an individual's upper body<br>* Distance to target: ranging from 1.4 to 9.1m (1.5 to 10 yards)<br>* Handgun: 0.40 caliber Smith and Wesson<br>* Accuracy/performance: total score (shots close to the center-of-mass or to the head: thee points; shots on vital areas of the upper body region but outside the center-of-mass: two points; shots on extremities: one point; max score: 30 points)

REFERENCE	COUNTRY	INTERVENTION	MARKSMANSHIP ASSESSMENT
Nieuwenhuys & Oudejans 2010 [27]<br><br>Effects of anxiety on handgun shooting behavior of police officers: a pilot study	The Netherlands	N/A	* Marksmanship course: 10 trials under LA and HA conditions. After instructed, officers fired 1 round at the opponent's right leg target, made a step to the right, fired 1 round at the opponent's left leg target, reloaded their handgun, fired 1 round at the opponent's chest target, stepped back to the left and fired another round at the chest target<br>* Target: opponent fitted with white target areas (one on the chest: 28 cm x 28 cm, and two on the upper legs: 12 cm x 35 cm).<br>* Distance to target: 5 m<br>* Handgun: Walther P5 9 mm (service weapon)<br>* Accuracy/performance: mean percentage of hits for each condition
Nieuwenhuys & Oudejans 2011 [28]<br><br>Training with anxiety: short- and long-term effects on police officers' shooting behavior under pressure	The Netherlands	* Four training sessions of 1 h consisting of several shooting exercises at the police academy's training facilities (e.g., a police car, a building, and a shopping street)<br># EG: practiced under additional pressure against an opponent that occasionally fired back<br># CG: practiced without additional pressure shooting at a mannequin or cardboard targets	* Marksmanship course: 10 trials under LA and HA conditions. After instructed, officers fired 1 round at the opponent's right leg target, made a step to the right, fired 1 round at the opponent's left leg target, reloaded their handgun, fired 1 round at the opponent's chest target, stepped back to the left and fired another round at the chest target<br>* Target: opponent fitted with white target areas (one on the chest: 28 cm x 28 cm, and two on the upper legs: 12 cm x 35 cm).<br>* Distance to target: 5 m<br>* Handgun: Walther P5 9 mm (service weapon)<br>* Accuracy/performance: mean percentage of hits for each condition
Oron-Gilad et al. 2008 [29]<br><br>The workload and performance relationship in the real world: a study of police officers in a field shooting exercise	USA	N/A	* Marksmanship course: four different night shooting tasks:<br># Warmup task: 16 rounds in very low ambient light conditions in which the target was barely visible<br># Flashlight task: 24 rounds while holding a flashlight. Some portions of the task required officers to shoot in very low ambient light conditions, whereas at other points officers used their flashlights<br># Barrel task: five targets and barrels were positioned along the shooting range target lane. The barrels were equally distributed over 27 m (approximately 6 m between two adjacent barrels). Officers moved

REFERENCE	COUNTRY	INTERVENTION	MARKSMANSHIP ASSESSMENT
			sequentially from barrel to barrel and shot three rounds as quickly as possible. On barrels 1, 2, and 5, officers reached the barrel and fired immediately. On barrels 2 and 4, officers took cover behind the barrel and illuminated the target with a flashlight prior shooting<br># Metal task: conducted in a different area of the shooting range illuminated by the strobe lights of a police cruiser. Officers aimed at one of three (for the first shooting line) or four (for the second shooting line) possible targets. At the first firing line, officers aimed at the targets and an instructor called out a color. Officers then had to hit the metal target of that color twice consecutively. This procedure was repeated twice. Once completed, the officer proceeded to the second firing line. Then, the instructor called out a second color and officers had again to shoot and hit the target twice consecutively. This procedure was also repeated twice<br>* Target:<br># Warmup, flashlight, and barrel tasks: standard paper target of a blue silhouette of a human figure against a white background<br># Metal task: square metal targets of three different colors distributed across two firing lines at two different distances. Unlike the paper silhouettes, the metal target provided auditory feedback after each shot as a result of the sound of the bullet hitting the metal target. Also, the metal targets were smaller than the paper targets and the officers were not familiar with them<br>* Distance to target: NR<br>* Handgun: SIG Sauer p226 9 mm<br>* Accuracy/performance: percentage of hits
Orr et al. 2018 [30]<br><br>The perception of the impact of load carriage on marksmanship performance in specialist police	Australia	Addition of tactical load (mean weight 23.5 kg ± 2.8 kg)	* Marksmanship course: five rounds at a self-selected pace in two different scenarios under both unloaded and loaded conditions:<br># Short forward movement: after instruction, officers commenced moving towards the target (short, forward movement) while shooting, staring at 10 m and concluding at 5 m from the target

REFERENCE	COUNTRY	INTERVENTION	MARKSMANSHIP ASSESSMENT
			# Mobility task: prior shooting, officers covered a total distance of 50 m, initially sprinting along a 30-m corridor, then performing a tactical clearance through three doorways, and finishing descending seven stairs. Officers then moved across the firing range, engaging the target at the 10-m line<br>* Target: human-shaped paper target with a 25 mm aiming circle positioned at center-of-mass<br>* Distance to target: ranging from 5 to 10 m<br>* Handgun: Glock<br>* Accuracy/performance: DCOT, horizontal dispersion (X), vertical dispersion (Y)
Orr et al. 2017 [7]<br><br>Grip Strength and Its Relationship to Police Recruit Task Performance and Injury Risk: A Retrospective Cohort Study	Australia	N/A	* Marksmanship course: 30 rounds over several trials. Officers stood square on to the target, with feet shoulder width apart and toes level (isosceles stance), with both arms fully extended towards the target and the pistol gripped firmly in both hands<br>* Target: Z-4 police target<br>* Distance to target: NR<br>* Handgun: Glock<br>* Accuracy/performance: total score (zero points awarded for a miss and one to four points per hit; pass score: 80 points)
Orr et al. 2021 [31]<br><br>Effect of grip size and grip strength on pistol marksmanship in police officers: A pilot study	Australia	N/A	* Marksmanship course: 10 rounds from a static, standing, unsupported position<br>* Target: Z-4 police targets with a center aiming point marked by a disc 70 mm in diameter<br>* Distance to target: 7 m<br>* Handgun: Glock 17<br>* Accuracy/performance: DCOT, horizontal displacement (X) and vertical displacement (Y)

REFERENCE	COUNTRY	INTERVENTION	MARKSMANSHIP ASSESSMENT
Oudejans 2008 [32]<br><br>Reality-based practice under pressure improves handgun shooting performance of police officers	The Netherlands	* Three 1-h training sessions over two weeks:<br># EG: trained shooting at each other<br># CG: trained shooting at a cardboard or other non-threatening targets	* Marksmanship course:<br># HP trial 1 (10 trials): officers stood in front of the opponent ready to use a pepper spray. At the starting signal, officers backed up shouting to the opponent to drop the weapons. When the distance between them was between 5 to 6 m, the opponent drew the knife, which was the signal for officers to drop the pepper spray, draw their handgun and take one shot at the leg targets of the opponent. The participant then kept withdrawing. At about 7 to 8 m, the opponent drew the handgun and aimed at the officers. This was the sign for officers to take a shot at the chest target of the opponent<br># HP trial 2 (10 trials): officers and opponent stood in front of a wall with their backs against each other. The opponent was facing the wall holding a handgun. At the start signal, officers walked away from the opponent while the opponent slowly turned around. When officers were between 5 to 7 m from the opponent, there was a whistle signal to sign officers to turn around, draw the gun and take one shot at the chest target<br># LP trials 1 and 2: similar to the HP trials except that officers shot at cardboard targets with whistle signals to notify officers when to shoot<br>* Target:<br># LP: life-size cardboard (125 cm by 70 cm) with one target at the chest (29 cm x 44 cm) and two at the legs (25 cm x 50 cm) and<br># HP: opponent fitted with plastic target areas, one on the chest (30 cm x 42.5 cm) and two at the legs (15 cm x 42.5 cm)<br>* Distance to target: ranging from 5 to 8 m<br>* Handgun: Walther P5 9 mm (duty weapon)<br>* Accuracy/performance: number of hits

REFERENCE	COUNTRY	INTERVENTION	MARKSMANSHIP ASSESSMENT
Thomas et al. 2018 [33]<br><br>Effect of Load Carriage on Tactical Performance in Special Weapons and Tactics Operators	USA	* Addition of tactical gear (mean weight 14.2 ± 2.0 kg):<br># Helmet<br># Chest worn tactical vest (ballistic armor, communications equipment, biceps protection, and 2 AR-15 magazines)<br># Tactical belt secured at the waist (Glock 35, handcuffs, and gas mask)	* Marksmanship course: STT under loaded and unloaded conditions: stair climb (18 stairs) / 3.2-m run around a door frame / descend the same flight of stairs / 44-m run / 1.5-m wall climb / 4.6-m walk / pick up an AR rifle / 5-round shooting with AR / 14-m walk / 10-round shooting with AR / 14-m sprint / 10-m run / 90° turn at a cone / 10-m run / 5.5-m walk / 4-m low crawl (army crawl) under a 0.6 m obstacle / 17-m walk / 5-round shooting with AR / 33-m run / drop the AR / pick up and carry a 19.1-kg battery ram for 30.8 m / door breach / drop the battery ram / 8.2-m walk / handgun firing at 6 targets<br>* Target: circular steel targets (diameter: 20.3 cm)<br>* Distance to target: 10 m<br>* Handgun: Glock 35, 0.40 caliber<br>* Accuracy/performance: number of rounds to knock down the 6 targets

Abbreviations: AR, Automatic rifle; AU, Arrest Unit; BC48, British Columbia 48-shot Firearms Course; CG, Control Group; cm, centimeter; DAO, double-action only; DCOT, Distance to Centre of Target; EEI, Execution-Emotion Imagery; EG, Experimental Group; EI, Execution Imagery; EW, emWave2; HA, High Anxiety; HP, High Pressure; HR, Heart Rate; HR_{max}, Maximum Heart Rate; HT, High Threat; kg, kilogram; LA, Low Anxiety; lb, pound; LP, Low Pressure; LT, Low Threat; m, meter; min, minute; mm, millimeter; N/A, Not Applicable; NR, Not Reported; PLA, placebo group; PPQ, Police Pistol Qualification; sec, second; STT, Simulated Tactical Test; USA, United States of America.
